# Neck circumference is associated with non-traditional cardiovascular risk factors in individuals at low-to-moderate cardiovascular risk: cross-sectional analysis of the Brazilian Longitudinal Study of Adult Health (ELSA-Brasil)

**DOI:** 10.1186/s13098-018-0388-4

**Published:** 2018-11-20

**Authors:** B. Almeida-Pititto, I. T. Silva, A. C. Goulart, M. I. H. Fonseca, M. S. Bittencourt, R. D. Santos, M. Blaha, S. Jones, P. P. Toth, K. Kulakarni, P. A. Lotufo, I. M. Bensenor, S. R. G. Ferreira, the ELSA Research Group

**Affiliations:** 1 Department of Preventive Medicine, Federal University of Sao Paulo, Rua Botucatu 740, São Paulo, SP 04023900 Brazil; 2 School of Public Health, University of São Paulo, Av. Dr. Arnaldo, 715, São Paulo, SP 01246-904 Brazil; 3 Department of Internal Medicine, University of São Paulo, Av. Lineu Prestes 2565, 4th Floor, São Paulo, SP 05508-000 Brazil; 4 Center for Clinical and Epidemiological Research, Hospital Universitário, University of São Paulo, Av. Lineu Prestes 2565, 3rd Floor, São Paulo, 05508-000 Brazil; 5 Lipid Clinic Heart Institute (InCor), University of Sao Paulo, Medical School Hospital, Av. Dr. Enéas de Carvalho Aguiar 44, São Paulo, 01246-000 Brazil; 6 Johns Hopkins Ciccarone Center for the Prevention of Heart Disease, Blalock 524 D1, 600 N. Wolfe St, Baltimore, MD USA; 7 Department of Preventive Cardiology, CGH Medical Center, 100 E. Le Fevre Road, Sterling, IL 61081 USA; 8 VAP Diagnostics Laboratory, 201 London Pkwy, Birmingham, AL 35211 USA

**Keywords:** Neck circumference, Cardiovascular risk factors, Non-traditional risk factors, Adipocytokines, E-Selectin, Lipoprotein subfractions

## Abstract

**Background:**

Neck circumference (NC) is associated with traditional cardiovascular risk factors (CVRF), but its usefulness to identify earlier atherogenic risk has been scarcely examined. Associations of NC with non-traditional CVRF were investigated in participants at low-to-moderate risk from the Brazilian Longitudinal Study of Adult Health (ELSA-Brasil).

**Methods:**

807 individuals (35–54 years) without obesity, diabetes or cardiovascular disease was stratified into quartiles of NC (cut-off for men: 36.5; 37.9 and 39.5 cm; women: 31.4; 32.5 and 34 cm) and traditional and non-traditional risk factors (lipoprotein subfractions by Vertical Auto Profile, adiponectin, leptin, E-selectin) were compared across groups. In linear regression models, associations of NC with non-traditional risk factors were tested for the entire sample and for low-risk group (≤ 2 CVRF).

**Results:**

In both sexes, BMI, waist circumference, systolic and diastolic blood pressure, fasting and 2-h plasma glucose, HOMA-IR, triglycerides, leptin, E-selectin, small dense LDL-cholesterol, IDL-cholesterol, VLDL_3_-cholesterol and TG/HDL ratio increased significantly, while HDL_2_-cholesterol and HDL_3_-cholesterol decreased across NC quartiles. In linear regression models, a direct association [β(95% CI)] of NC with leptin [(0.155 (0.068–0.242); 0.147 (0.075–0.220)], E-selectin [(0.105 (0.032–0.177); 0.073 (0.006 to 0.140)] and small-dense LDL [(1.866 (0.641–3.091); 2.372 (1.391–3.353)] and an inverse association with HDL_2_-cholesterol [(− 0.519 (− 0.773 to − 0.266); − 0.815 (− 1.115 to 0.515)] adjusted for age were detected for men and women, respectively.

**Conclusion:**

Our findings indicate that measurement of NC may be useful for an earlier identification of unfavorable atherogenic metabolic profile in middle-aged individuals at lower cardiovascular risk level.

## Introduction

Cardiovascular disease (CVD) is the leading cause of disability adjusted life years (DALYs) worldwide, particularly in developing countries [1]. Earlier identification of at-risk individuals using novel risk markers could anticipate the implementation of preventive strategies.

Considering the role of insulin resistant adipose tissue in atherogenesis, measuring accurate adiposity indicators of cardiometabolic risk are clinically informative. Beyond the usefulness of body mass index (BMI) and waist circumference (WC), there is some evidence that neck circumference (NC) could also reflect upper-body fat deposition, enhancing the identification of high-risk individuals [2]. Increased NC has been reported in association with sleep apnea, elevated blood pressure, insulin resistance, lipid abnormalities and metabolic syndrome [3]. More recently, in studies including high-risk or diabetic individuals, NC was also associated with C-reactive protein (CRP), uric acid and carotid intimal-media thickness [4, 5]. How NC could help predicting cardiometabolic risk earlier has not been adequately investigated in large studies. The relationship between NC and non-traditional cardiovascular biomarkers in non-obese individuals without overt CVD warrants further investigation.

Great debate exists regarding the utility of circulating biomarkers of inflammation and endothelial dysfunction [6] and of atherogenic lipoprotein subfractions, which are increased in obesity prior to the development of overt type 2 diabetes or cardiovascular events [7]. Since these biomarkers play pathophysiological roles, they may represent an opportunity to identify risk earlier in the natural course of cardiometabolic diseases. E-selectin concentrations are associated with increased risk of diabetes mellitus [8] and calcium deposition in coronary arteries of low-to-moderate risk individuals [9]. Leptin and adiponectin are cytokines related to body adiposity and systemic inflammatory tone [10, 11]. Lipoprotein subfractions influence cardiovascular risk [12]. Increased serum concentrations of very low-density lipoproteins, remnant lipoproteins, small dense low-density lipoprotein (LDL), and decreased high-density lipoprotein (HDL) particles levels have been consistently associated with coronary heart disease [13, 17]. However, their predictive value for clinical practice is still not widely endorsed.

In this cross-sectional analysis of participants from the Brazilian Longitudinal Study of Adult Health (ELSA-Brasil) [14], we hypothesized that NC could identify an atherogenic profile based on determinations of non-traditional cardiovascular risk factors, namely adipocytokines (adiponectin and leptin), an endothelial adhesion molecule (E-selectin), and lipoprotein sub fractions in non-obese individuals at low-to-moderate cardiovascular risk.

## Methods

### Design and population study

ELSA-Brasil [15] is an ongoing prospective cohort that enrolled 15,105 civil servants aged 35–74 years (54% women) in Brazil and aims to investigate type 2 diabetes, CVD and their risk factors [16]. The present cross-sectional analysis was based on the baseline data (carried out from August 2008 through December 2010) from a random sample of 1000 out of 5061 participants of the São Paulo research center included in a sub study aimed to evaluate the cardiometabolic profile based on non-traditional cardiovascular biomarkers (inflammatory and endothelial dysfunction biomarkers). The inclusion criteria were age range of 35–54 years and absence of diabetes (self-reported diabetes plus use of hypoglycemic drug or diabetic diagnosis by oral glucose tolerance test) and self-reported CVD. For the current analysis, obese individuals were excluded (BMI > 30 kg/m^2^). The sample was then composed of 807 participants. The institutional ethics committee approved the study and written consent was obtained from all participants.

### Traditional CVRF

Body weight and height were measured using calibrated electronic scales and a fixed rigid stadiometer, while individuals wore light clothing without shoes. BMI was calculated as weight (in kilograms) divided by squared height (in meters). Waist circumference was measured with an inextensible tape according to the World Health Organization technique. NC was measured with individuals sitting and looking horizontally, using an inelastic tape, perpendicular to the long axis of the neck, right under the thyroid cartilage. Blood pressure was taken three times after a 5-min rest in the sitting position and the mean between the second and third measurements was used [16].

Participants underwent a 2-h 75-g oral glucose tolerance test (OGTT) for diagnosing categories of glucose tolerance [16]. Insulin resistance was estimated using the HOMA-IR index: fasting insulin (µUI/mL) × fasting glucose (mmol/L)/22.5 [17]. Plasma glucose was determined by the hexokinase method (ADVIA Chemistry; Siemens, Deerfield, Illinois, USA). ELISA kits were used for the determination of insulin (Siemens, Tarrytown, USA). Glomerular filtration rate (GFR) was estimated using the formula of Chronic Kidney Disease Epidemiology Collaboration—CKD-EPI [18].

Participants were categorized according to the presence of the following cardiovascular risk factors: (1) WC ≥ 102 cm for men or ≥ 88 cm for women; (2) systolic or diastolic blood pressure ≥ 130/85 mmHg or antihypertensive treatment; (3) fasting plasma glucose ≥ 100 mg/dL and < 126 mg/dL in the absence of antidiabetic agents; (4) triglyceride ≥ 150 mg/dL, or specific treatment; (5) HDL-C < 40 mg/dL for men and < 50 mg/dL for women, or specific treatment [20]. Those who had up to 2 abnormalities were considered at lower risk for cardiovascular disease and those with 3 or more of these cardiovascular risk factors were considered as having a higher cardiovascular risk.

### Non-traditional CVRF

Aliquots were frozen at − 80 °C for further determinations of adipocytokines and lipid subfractions [16]. ELISA kits were used for the determination of adiponectin, leptin and E-selectin (Enzo Life Sciences, Farmingdale, NY, USA).

Lipid profiles were characterized by VAP testing (Atherotech, Birmingham, AL, USA), which is an inverted rate zonal, single vertical spin, density gradient ultracentrifugation method to separate lipoproteins into their subclasses [19]. This technique directly measures the total cholesterol in LDL real cholesterol (LDLr-C) and LDL subfractions (LDL-C_1–4_); VLDL-C (very low-density lipoprotein cholesterol) (VLDL-C_1+2_ and VLDL_3_-C); IDL-C (intermediate density lipoprotein); total HDL-C and its subfractions (HDL_2_-C and HDL_3_-C); and lipoprotein (a).

Here, we evaluated T-Cholesterol and its subfractions: LDL-C (real LDL-C + IDL-C + Lp (a)-C), IDL-C and the real LDL (LDL_r_-C), which is biochemically defined by LDL-C fraction from the ultracentrifugation separation of the lipids by VAP. In addition, the following subclasses were evaluated: small dense LDL-C (LDL_3_-C + LDL_4_-C); larger buoyant LDL-C (LDL_1_-C + LDL_2_-C); VLDL_3_-C (small dense cholesterol-rich VLDL subfraction); non-HDL-C (non-high-density lipoprotein cholesterol) that corresponds to a sum of LDL_r_-C, VLDL-C, IDL-C and Lp (a) was analyzed; HDL-C and its sub-fractions HDL_2_-C (larger, buoyant subclass) and HDL_3_-C (smaller, denser subclass). Finally, we calculated the logarithm of LDL-C density ratio [LLDR, ln ((LDL_3_-C + LDL_4_-C)/(LDL_1_-C + LDL_2_-C))], which is closely related to ultracentrifugation-derived LDL density phenotype [18]. Total plasma triglycerides were measured by an enzymatic colorimetric assay (ADVIA 1200, Siemens, Calif., USA).

### Statistical analyses

Data were expressed as mean and standard deviation (SD) or as median and interquartile range (IQR) according to continuous variables distribution. Individuals were stratified into quartiles of neck circumference (Men: first quartile, Q1, < 36.5 cm; second quartile, Q2, 36.5 to < 37.9 cm; third quartile, Q3, 37.9 to < 39.5 cm; forth quartile, Q4, ≥ 39.5 cm. Women: first quartile, Q1, < 31.4 cm; second quartile, Q2, 31.4 to < 32.5 cm; third quartile, Q3, 32.5 to < 34 cm; fourth quartile, Q4, ≥ 34 cm). Continuous and categorical variables were compared across NC quartiles using ANOVA and the Chi square test, respectively.

Multiple linear regression models were built to test the associations of NC with biomarkers, lipids and its subfractions, adjusted for age, in total sample, and according to the low-risk group. To evaluate the behavior of this association in individuals at different levels of cardiovascular risk, the linear regression analysis was also stratified by the presence of up to 2 cardiovascular risk factors. Statistical analyses were performed using the Statistical Package for Social Sciences, version 19.0 for Windows (SPSS Inc., Chicago, Illinois, USA). A *p* value < 0.05 was considered significant.

## Results

Of the 807 individuals, 441 were women. Table 1 shows the comparison of mean (SD) or median (IQR) of the risk factors and subfractions of cholesterol across the NC quartiles according to sex. In both sexes, most cardiovascular risk factors such as WC, BMI, systolic and diastolic blood pressure, seric creatinine, fasting and 2-h plasma glucose, HOMA-IR triglycerides increased gradually across the NC quartiles but total cholesterol and total and real LDL-cholesterol (Table 1). HDL-C levels and estimated glomerular filtration rate fasting and 2-h plasma glucose, HOMA-IR triglycerides increased gradually across the NC quartiles but not total cholesterol and total and real LDL-cholesterol (Table 1). HDL-C levels were inversely associated with NC quartiles in both sexes with borderline significance. Leptin, E-selectin, small dense LDL-C, IDL-C, VLDL_3_-C and TG/HDL ratio increased, and HDL_2_-C and HDL_3_-C decreased, while adiponectin, large LDL and log LDL-DR did not differ across the quartiles.

**Table 1:** Characteristics of participants stratified according to sex and neck circumference

	Men N = 366					Women N = 441				
	Q1 n = 89	Q2 n = 93	Q3 n = 86	Q4 n = 98	p-value	Q1 n = 104	Q2 n = 106	Q3 n = 112	Q4 n = 119	p-value
Age (years)	45 (5)	45 (5)	45 (4)	46 (5)	0.789	45 (5)	46 (4)	46 (5)	46 (5)	0.320
BMI (kg/m^2^)	22.3 (2.4)	24.7 (2.3)	26.1 (2.3)	27.4 (1.6)	< 0.001*	22.3 (2.2)	24.2 (2.5)	25.2 92.0)^Ω ¥^	26.7 (2.1)^Ω ¥ π^	< 0.001
Waist circumference (cm)	80.5 (7.3)	86.3 (6.5)^Ω^	89.9 (6.6)^Ω ¥^	94.5 (5.2)^Ω ¥ π^	< 0.001*	73.7 (6.0)	78.2 (6.8)	80.9 (6.0)^Ω ¥^	85.0 (6.3)^Ω ¥ π^	< 0.001*
Systolic BP (mmHg)	118 (12)	119 (12)	123 (15)	125 (13)^Ω^	0.002*	108 (12)	110 (13)	112 (13)	113 (12)^Ω^	0.014*
Diastolic BP (mmHg)	74 (9)	77 (10)	77 (10)	81 (9)^Ω¥^	< 0.001*	69 (9)	71 (9)	72 (9)	73 (9)^Ω^	0.005*
Creatinine (mg/dL)	1.02 (0.15)	1.09 (0.15)	1.05 (0.15)	1.11 (0.42)	0.035	0.78 (0.11)	0.81 (0.12)	0.85 (0.14)	0.84 (0.13)	< 0.001*
Glomerular filtration rate (GFR)	90.3 (13.9)	84.6 (14.4)	89.4 (15.2)	86.0 (17.2)	0.073	94.1 (14.5)	89.8 (15.3)	86.7 (16.5)	87.3 (15.4)	0.002*
Fasting glucose (mg/dL)	102.8 (7.9)	103.8 (7.9)	105.2 (8.1)	105.6 (8.3)	0.064*	97.8 (6.4)	99.3 (7.8)	100.9 (7.2)^Ω^	100.9 (7.2)^Ω^	0.004*
2-h glucose (mg/dL)	11.6 (6.5)	115.6 (25.5)	121.5 (29.6)	123.1 (26.9)^Ω^	0.016*	112.4 (24.0)	116.8 (22.8)	118.2 (21.6)	123.3 (26.0)^Ω^	0.009*
HOMA-IR	0.8 (0.3 to 1.5)	1.3 (0.9 to 2.1)	1.7 (0.1 to 2.4)	1.9 (1.3 to 2.9)	< 0.001*	0.9 (0.5 to 1.5)	1.1 (0.7 to 1.7)	1.3 (0.7 to 2.0)	1.7 (1.1 to 2.5)	< 0.001*
Leptin (ng/mL)	5.6 (2.3 to 10.7)	6.7 (3.9 to 12.6)	7.2 (4.8 to 11.4)	7.9 (5.3 to 12.1)	0.002*	12.4 (7.8 to 21.3)	15.2 (8.1 to 24.3)	17.6 (10.6 to 29.1)	18.8 (12.1 to 30.1)	0.001*
Adiponectin (mcg/mL)	10.3 (6.2 to 15.1)	9.8 (5.8 to 13.7)	9.6 (6.5 to 13.4)	6.5 (4.0 to 12.2)	0.216	11.8 (7.6 to 16.8)	10.6 (6.0 to 15.2)	9.4 (5.6 to 13.9)	9.7 (4.9 to 15.5)	0.358
E-selectin (ng/mL)	61.1 (39.0 to 105.8)	77.8 (51.2 to 127.1)	80.5 (41.5 to 114.4)	87.8 (65.2 to 131.6)	0.006*	61.9 (38.5 to 92.8)	62.1 (37.5 to 99.8)	66.0 (38.2 to 114.2)	76.4 (47.6 to 120.5)	0.162*
Total cholesterol^#^ (mg/dL)	211 (187 to 235)	206 (182 to 231)	219 (188 to 239)	218 (194 to 243)	0.156	206 (185 to 238)	215 (192 to 238)	207 (186 to 242)	213 (189 to 143)	0.761
Non HDL-cholesterol^#^ (mg/dL)	159 (128 to 181)	160 (132 to 179)	165 (137 to 185)	168 (146 to 192)	0.001*	144 (123 to 169)	155 (129 to 171)	149 (126 to 178)	155 (131 to 188)	0.140
HDL-cholesterol^#^ (mg/dL)	53.0 (45 to 62)	48 (42 to 54)	47 (40 to 55)	46 (41 to 53)	0.059*	63 (57 to 70)	60 (52 to 69)	58 (49 to 66)	54 (47 to 68)	< 0.001*
LDL-total^#^ (mg/dL)	138 (111 to 149)	134 (104 to 151)	135 (111 to 158)	138 (116 to 157)	0.527	111 (102 to 145)	134 (107 to 147)	124 (107 to 152)	130 (107 to 156)	0.493
LDL R^#^ (mg/dL)	116 (92 to 129)	109 (86 to 125)	112 (88 to 132)	114 (93 to 129)	0.672	101 (82 to 122)	108 (85 to 124)	105 (88 to 125)	107 (90 to 130)	0.602
IDL-cholesterol^#^ (mg/dL)	14 (10 to 19)	15 (12 to 21)	17 (12 to 22)	18 (14 to 22)	0.003*	14 (10 to 18)	13 (11 to 20)	13 (9 to 18)	15 (11-23)	0.012
Triglycerides^#^ (mg/dL)	92 (71 to 127)	108 (86 to 140)	114 (89 to 174)	146 (96 to 193)	< 0.001*	76 (61 to 98)	84 (70 to 115)	82 (67 to 110)	106 (73 to 144)	< 0.001*
HDL_2_-C^#^ (mg/dL)	13.0 (10 to 17)	11 (8 to 14)	11 (8 to 15)	11 (9 to 13)	0.001*	19 (16 to 24)	18 (14 to 22)	16 (13 to 20)	15 (12 to 20)	< 0.001*
HDL_3_-C^#^ (mg/dL)	39 (34 to 46)	36 (33 to 40)	37 (31 to 41)	36 (32 to 41)	0.008	43 (40 to 48)	41 (38 to 47)	41 (37 to 46)	40 (36 to 46)	0.002
Large LDL-C^#^ (mg/dL)	40 (30 to 49)	39 (31 to 49)	40 (33 to 50)	39 (32 to 52)	0.763*	43 (30 to 61)	44 (31 to 58)	41 (29 to 55)	42 (33 to 59)	0.723
Small LDL-C^#^ (mg/dL)	53 (38 to 79)	60 (37 to 79)	62 (42 to 81)	72 (48 to 88)	0.060*	38 (29 to 49)	41 (31 to 54)	44 (32 to 61)	47 (33 to 64)	0.005*
Log LDL DR^#^ (mg/dL)	0.37 (0.02 to 0.73)	0.48 (0.09 to 0.72)	0.52 (0.13 to 0.73)	0.59 (0.17 to 0.81)	0.295	− 0.17 (− 0.60 to 0.36)	− 0.04 (− 0.41 to 0.33)	0.14 (− 0.31 to 0.58)	0.20 (− 0.27 to 0.53)	0.012*
VLDL_3_-C^#^ (mg/dL)	13 (10 to 15)	15 (12 to 18)	14 (13 to 17)	16 (13 to 20)	< 0.001	12 (9 to 14)	12 (11 to 15)	12 (10 to 14)	14 (11 to 17)	< 0.001
TG/HDL ratio	1.7 (1.2 to 2.7)	2.4 (1.7 to 3.3)	2.4 (1.6 to 4.0)	3.1 (2.0 to 4.5)	< 0.001	1.2 (0.9 to 1.6)	1.4 (1.0 to 2.0)	1.4 (1.1 to 2.2)	1.9 (1.2 to 2.7)	< 0.001

BMI, body mass index NC, neck circumference BP, blood pressure

Glomerular filtration rate (GFR) was estimated using the formula of Chronic Kidney Disease Epidemiology Collaboration—CKD-EPI

Lipid profile evaluated by Vertical Auto Profile

Values are means (SD) or medians (interquartile intervals)

Men: first quartile, Q1, < 36.5 cm; second quartile, Q2, 36.5 to < 37.9 cm; third quartile, Q3, 37.9 to < 39.5 cm; forth quartile, Q4, ≥ 39.5 cm. Women: first quartile, Q1, < 31.4 cm; second quartile, Q2, 31.4 to < 32.5 cm; third quartile, Q3, 32.5 to < 34 cm; forth quartile, Q4, ≥ 34 cm

p value, ANOVA was used. ^Ω^ versus Q1; ^¥^ versus Q2; ^π^ versus Q3. ^#^ Kruskal–Wallis test was used. * p for trend < 0.05

The frequency of central obesity defined by WC was 4.9% and 14.3%, hypertension 24.3% and 12.0%, hypertriglyceridemia 32.8% and 14.5%, low HDL-cholesterol levels 13.7% and 19.7% and pre-diabetes 75.1% and 54.2% in men and women, respectively. Those who had up to 2 traditional CVRF were considered at low-risk (83% men and 89% women). The prevalence of having 3 or more traditional CVRF increased across the groups of neck quartiles (Q1: 6.7% and 2.9%; Q2: 9.8% and 7.8%; Q3: 21.2% and 11.7%; Q4: 30.6% and 19.8%) in both men and women respectively. For each age-adjusted 1 cm increase in NC, changes of + 2.3 cm in waist circumference, + 0.85 kg/m^2^ in BMI, + 0.5 mg/dL in fasting glucose and + 0.9 mmHg in systolic blood pressure for men and women were observed.

**Fig. 1:**
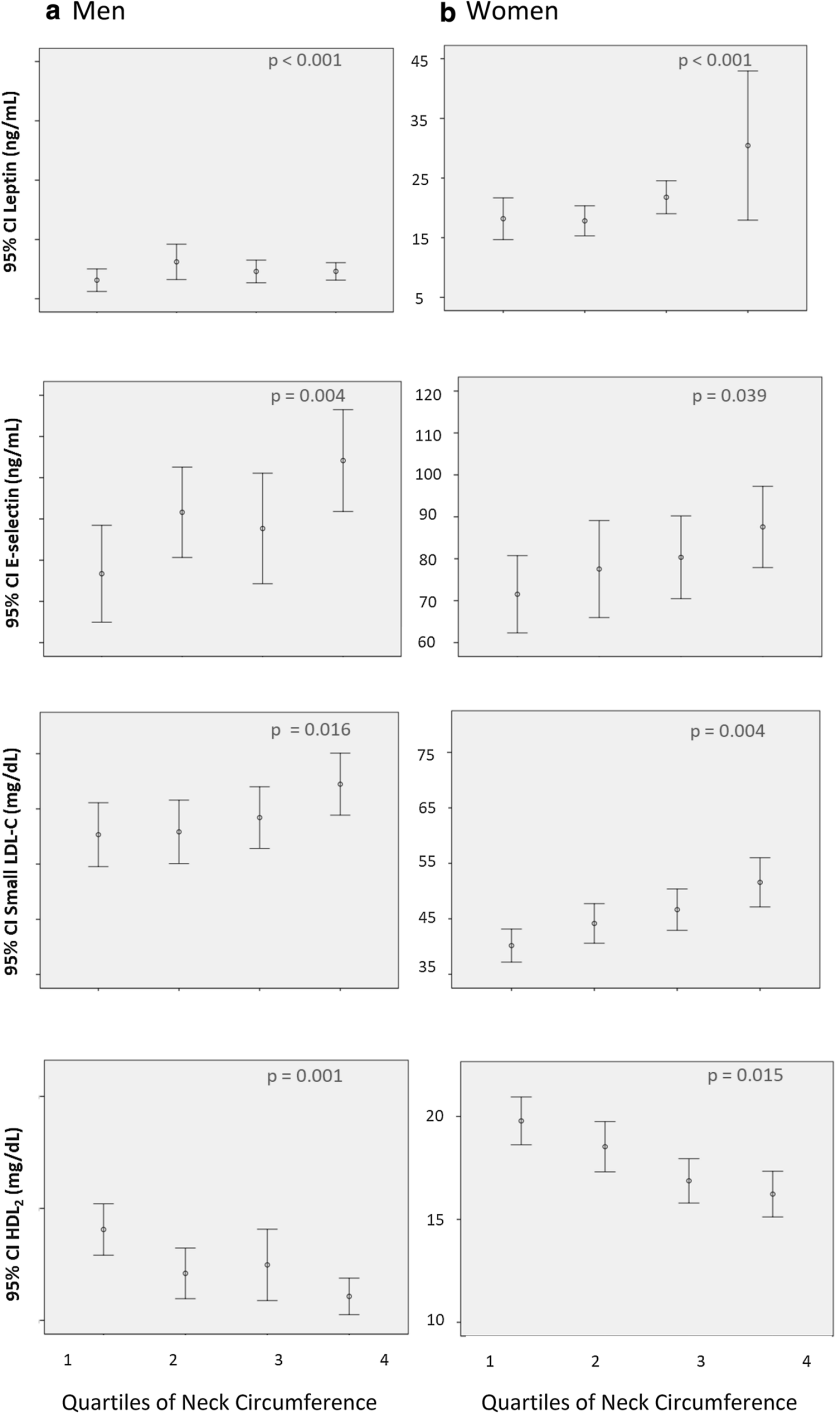
Mean (95% CI) values of leptin, E-selectin, small LDL-C, HDL2-C according to neck circumference in men and women

Biomarkers related to atherogenesis—leptin, E-selectin, small LDL and HDL2—increased across the NC quartiles for each sex are shown in Fig. 1. There is a tendency for worsening of the lipid profile according to an increase of NC was observed.

Due to the pattern observed for leptin, E-selectin, small-dense LDL and HDL_2_ concentrations across the NC categories, the association between each of these variables was examined in linear regression analysis. A direct and independent association of NC with leptin, E-selectin and small-dense LDL and an inverse independent association with adiponectin and HDL_2_ were detected for the entire sample and also for individuals of both sexes with ≤ 2 risk factors (Table 2). For the group of participants with ≥ 3 cardiovascular risk factors the results were not significant.

**Table 2:** Association of leptin, E-selectin and sub fractions of lipoprotein with neck circumference according to sex, considering the entire sample and individuals at low cardiovascular risk

	Leptin			E-selectin			Small-dense LDL-C			HDL_2_-C		
	β	95% CI	p	β	95% CI	p	β	95% CI	p	β	95% CI	p
Entire sample												
Men												
Neck circumference	0.155	0.069 to 0.242	< 0.001	0.105	0.032 to 0.177	0.005	1.866	0.641 to 3.091	0.003	− 0.519	− 0.773 to − 0.266	< 0.001
Women												
Neck circumference	0.147	0.075 to 0.220	< 0.001	0.73	0.006 to 0.140	0.032	2.372	1.391 to 3.353	< 0.001	− 0.815	− 1.115 to − 0.515	< 0.001
Lower risk subsample												
Men												
Neck circumference	0.183	0.084 to 0.282	< 0.001	0.95	0.015 to 0.175	0.021	1.758	0.478 to 3.039	0.007	− 0.476	− 0.766 to − 0.186	0.001
Women												
Neck circumference	0.152	0.075 to 0.229	< 0.001	0.068	− 0.004 to 0.140	0.066	1.620	0.597 to 2.643	0.002	− 0.685	− 1.013 to − 0.356	< 0.001
Higher risk subsample												
Men												
Neck circumference	0.001	− 0.103 to 0.103	0.995	0.074	− 0.033 to 0.181	0.173	− 2.520	− 6.299 to 1.260	0.187	0.165	− 0.273 to 0.640	0.454
Women												
Neck circumference	− 0.014	− 0.068 to 0.041	0.619	0.015	− 0.034 to 0.064	0.545	1.610	− 1.484 to 4.705	0.300	− 0.076	− 0.514 to 0.667	0.796

Linear regression analysis. Adjusted for age. Lower risk subsample—individuals with up to 2 cardiovascular risk factors. Higher risk subsample—3 or more cardiovascular risk factors. Traditional cardiovascular risk factors: (1) waist circumference ≥ 102 cm for men or ≥ 88 cm for women; (2) systolic or diastolic blood pressure ≥ 130/85 mmHg or antihypertensive treatment; (3) fasting plasma glucose ≥ 100 mg/dL and < 126 mg/dL in the absence of antidiabetic agents; (4) triglyceride ≥ 150 mg/dL, or specific treatment; (5) HDL-cholesterol < 40 mg/dL for men and < 50 mg/dL for women, or specific treatment

Sensitivity analyses, excluding participants under medications (antihypertensive and/or lipid reducing agents and/or hormone therapy), current smoking and menopause were performed but results did not change.

## Discussion

Our findings showed the ability of NC to identify a risk profile, including non-traditional biomarkers such as leptin, E-selectin and lipoprotein subfractions, in non-obese individuals, considered at low-to-moderate cardiovascular risk.

Despite the recognized impact of traditional cardiovascular risk factors such as increased age, hypertension, smoking, diabetes and abnormalities in lipoprotein metabolism [20], a considerable proportion of individuals without these factors sustain cardiovascular events. Considering the multiplicity of factors involved in atherogenesis, measurement of markers of endothelial dysfunction and insulin resistance could help identify subsets of patients at increased cardiovascular risk.

Some studies have already reported the association of NC with cardiovascular risk factors, namely central obesity, hypertension, insulin resistance, and dyslipidemia in individuals at higher cardiovascular risk [4, 21–23]. NC was suggested as a risk factor independent of adipose tissue mass and distribution [2]. Furthermore, NC was found to be a predictor of fatal and non-fatal cardiovascular events as well as of renal dysfunction in a sample of high-risk patients, [24, 25], but we are not aware of studies that have evaluated the role of NC in low risk individuals as we did. In the present study, even in non-obese individuals, increments in waist circumference, blood pressure, and plasma glucose values were observed across NC quartiles, while HDL-cholesterol and estimated glomerular filtration rate decreased. We emphasize that the mean values of these traditional cardiovascular risk factors, creatinine and estimated glomerular filtration rate, were within normal ranges.

A gradual increase of leptin and decrease of adiponectin levels were detected across the NC quartiles. Previous studies have shown that low adiponectin and high leptin levels were associated with a pro-inflammatory state, insulin resistance and coronary artery calcium severity in adults [4, 26]. It was suggested that NC could be a predictor of low-grade systemic inflammation in adults as it was reported in children [27]. Our findings support the assumption that NC could identify low-to-moderate risk individuals who already have a worse profile of adipocytokines related to insulin resistance and low grade inflammation.

Leukocyte-endothelial cell adhesion molecules, such as E-selectin, are also expressed in response to cytokines and play a role in the atherogenesis [28]. Interestingly, we found a linear increase in E-selectin concentrations in parallel to increases in NC as shown in Fig. 1 (p for trend = 0.039 for women and 0.004 for men). In a previous analysis of the ELSA-Brasil, we reported that E-selectin was associated with insulin resistance and the presence of calcium in coronary arteries in individuals without diabetes or cardiovascular disease [29]. We proposed that higher circulating E-selectin levels, found across the quartiles of NC, might be suggestive of early atherogenesis, as well as of an early disturbance of glucose metabolism.

We used the VAP measurements to provide information beyond the basic lipid profile and may help identify individuals at higher cardiovascular risk. Small dense LDL are biophysically more likely to access the subendothelial space and more prone to oxidation. It is known that hypertriglyceridemia and low HDL-C are associated with a predominance of small dense LDL-C (LDL_3_-C and LDL_4_-C) particles (also known as phenotype B) [30, 31]. Increased hepatic lipase activity and triglyceride enrichment of lipoproteins are commonly found in states of insulin resistance, resulting in a reduction of HDL-C—predominantly the HDL_2_-C subclass—and a relative or absolute increase in the small dense HDL_3_-C [31, 32]. This scenario accelerates atherogenesis, contributing to elevate cardiovascular risk. In this context, NC was able to indicate similar lipoprotein subfractions alterations (HDL_2_-C reduction, TG/HDL ratio elevation) that are strongly associated with insulin resistance and small LDL. In addition, it was directly associated with small-dense LDL-C and negatively with HDL_2_-C, maintaining significance even when including only low risk individuals. Therefore, we hypothesize that NC might be a useful proxy of early lipid profile disturbances involved in atherogenesis.

It is well known the importance of other anthropometric measurements, such as BMI and waist circumference, to predict cardiometabolic risk and the actual study provide evidence that NC could be another anthropometric measurement to identify early atherogenic profile. To emphasize the usefulness of NC, this study evaluated a sample constituted by non-obese individuals (BMI up to 30) and regression analysis were stratified according to the number of CV risk factors. Waist circumference did not enter in final models of multiple regression analysis to avoid over adjustments, since NC is associated with waist and both may represent the association between adiposity and cardiometabolic risk. Comparing to waist circumference, neck circumference might be an easier performed anthropometric measurement for clinical practice.

The cross-sectional design of this study limits concluding that increased NC is causal for the associations described herein. Increased NC is highly correlated with increased insulin resistance, a driving force for many of the metabolic alterations we found. Significant associations of NC with non-traditional risk factors were not detected in our linear regression analyses, and two possible explanations were considered: the number of individuals with 3 or more cardiovascular risk factors (63 men and 47 women) could be not sufficient to reach statistical significance. Also, we could hypothesize that the risk of individuals with ≥ 3 major risk factors is already so high that elevated non-traditional risk factors do not differ among the NC quartiles anymore.

Strengths of our study were its large sample of low-moderate risk individuals and the analysis of novel circulating markers of initial arterial damage. The follow-up of these ELSA-Brasil participants should allow us to test the hypothesis raised in this present study.

In conclusion, this study verified that non-obese individuals with higher neck circumference demonstrate more abnormalities in traditional risk factors and non-traditional risk factors such as leptin, adiponectin and selectin as well as a more atherogenic lipid profile even in a low-risk group. Neck circumference is an easily performed anthropometric measurement that may potentiate early identification of those individuals at low-to-moderate risk in whom markers of atherogenesis are readily detected.
